# MRI Findings in Hip in Juvenile Idiopathic Arthritis

**DOI:** 10.3390/jcm10225252

**Published:** 2021-11-11

**Authors:** Monika Ostrowska, Piotr Gietka, Małgorzata Mańczak, Emil Michalski, Iwona Sudoł-Szopińska

**Affiliations:** 1 Department of Radiology, National Institute of Geriatrics, Rheumatology and Rehabilitation, 02-637 Warsaw, Poland; monique.ostrowska@gmail.com (M.O.); sudolszopinska@gmail.com (I.S.-S.); 2 Clinic of Paediatric Rheumatology, National Institute of Geriatrics, Rheumatology and Rehabilitation, 02-637 Warsaw, Poland; malgieta1@gmail.com; 3 Department of Gerontology, Public Health and Didactics, National Institute of Geriatrics, Rheumatology and Rehabilitation, 02-637 Warsaw, Poland; m.manczak@op.pl

**Keywords:** arthritis, juvenile, hip, arthralgia, magnetic resonance imaging

## Abstract

The aim of this study was to evaluate if magnetic resonance imaging allows hip arthritis in JIA to be differentiated from hip arthralgia of unknown etiology in juveniles clinically suspected for hip arthritis. This was a retrospective observational study which included 97 children with clinically suspected hip arthritis. Each hip was assessed and scored in MRI for signs of active and destructive inflammatory lesions and developmental lesions. MRI findings between JIA-confirmed patients and without final diagnosis of JIA were compared and the MRI summarized score was calculated, as the sum of scorings of all 24 hip lesions in an individual patient (i.a., effusion, synovitis, bone marrow edema, enthesitis). MRI showed at least one lesion in the majority of patients (95 patients; 98%). Effusion was the most common feature, followed by bone marrow oedema and synovitis. All lesions were more common in patients with a final diagnosis of JIA, especially synovitis and enthesitis (*p* = 0.037 and *p* = 0.047). The MRI summarized score was significantly higher in the JIA group than the non-JIA group: 3 (2–5) vs. 2 (2–2), respectively, *p* = 0.002. Using a cut-off score of 6, the MRI summarized score showed 25% sensitivity and 100% specificity indicating a good ability in discriminating hip arthritis during JIA from non-JIA patients. MRI allows hip arthritis in JIA to be differentiated from hip arthralgia of unknown etiology with good specificity, thus, may be helpful in confirming the diagnosis of JIA.

## 1. Introduction

Juvenile idiopathic arthritis (JIA) is a heterogeneous group of diseases with an onset before 16 years of age [1,2]. It typically lasts for more than six months, with arthritis present for at least six weeks [1,2] and is the most common form of childhood arthritis [3,4,5,6,7]. Joint involvement usually starts with synovitis and the formation of inflammatory tissue, called the pannus, which destroys hyaline cartilage, erodes the bone and leads to articular destruction and ankylosis [5,6]. As highly effective treatment is now available for treating JIA, there is increasing demand for novel imaging techniques to provide objective and accurate measures of inflammatory changes to monitor the disease and treatment response [8].

Hip arthritis develops in 20–63% of children with JIA, mainly in juveniles with the systemic subtype of the disease [1,4,5,9,10,11] and is often a predictor of severe disease and high-risk disability [1]. The clinical features are not specific for active hip inflammation and may occur due to previous joint damage [11] or other hip diseases, including malignancies. Furthermore, since it is not a superficial joint, hip involvement is particularly difficult to detect clinically, as inflamed synovium and effusions cannot be directly palpated [4,12].

Magnetic resonance imaging (MRI) is a sensitive and valuable technique in pediatric musculoskeletal pathologies and is considered to be the most suitable technique for detecting synovial hypertrophy and bone marrow oedema (BME) [13,14,15,16,17,18]. It allows the visualization of joints in several dimensions using a multiplanar technique [4]. The most common pathologies in JIA are effusions and synovitis, which are most accurately diagnosed using gadolinium injection, following which the highly vascular pannus enhances brightly while joint effusion remains of low signal [19]. Recent studies have highlighted potential adverse effects of gadolinium [20,21]. Consequently, due to observed gadolinium depositions in the brain, the European Medicines Agency (EMA) has now banned several linear gadolinium-based contrast agents. Moreover, even though macrocyclic contrast agents have not been suspended, they should still be used with care and a strict indication [20]. The new term “effusion/synovial thickening” was used lately by Panwar et al. [22] defined as “an increased amount (greater than physiologic) of high signal intensity within the joint space distending the joint capsule on T2-weighted fat—saturated or STIR or fluid sensitive sequences”. “Effusion/synovial thickening” was considered altogether as a single item as it is often difficult to differentiate these two findings by MRI without intravenous administration of contrast [22]. In addition to synovitis and osteitis, other inflammatory lesions that may be seen in children with JIA include bursitis, enthesitis and myositis [23,24,25,26]. Inflammation may also involve the triradiate cartilage and growth plates. Destructive, irreversible lesions in the hip joint include cysts, erosions, joint space narrowing, chondromalacia and ankylosis. Such joint inflammation that interferes with bone growth may eventually lead to skeletal growth disturbances and developmental disorders, such as bone remodeling.

Several scoring systems for juvenile hip arthritis assessment on MRI have been proposed [4,5,11,27,28]; however, none of them includes all the above-mentioned items in JIA (i.e., active, destructive and developmental) and, to date, a validated MRI scoring system for assessing the hips in JIA has not been established [29]. An important methodological work on standardizing the whole-body-MRI scoring system, including the hip joint, for assessment of disease activity in JIA was published in 2021 by the MRI in JIA OMERACT working group [22].

Understanding limitations of clinical assessment of hip joint arthritis, the aim of this study was to investigate if MRI allows hip arthritis in JIA to be differentiated from hip arthralgia of unknown etiology.

## 2. Materials and Methods

### 2.1. Patients

This was a retrospective single center study which included 97 children aged 4–16 years with clinically suspected hip disease in JIA [30,31]. The study was based on the analysis of data available in the hospital database. All children meeting the inclusion criteria hospitalized in the period from 2016 and 2019 were included in the analysis.

Reported complaints included hip pain at rest or on movement, or restricted movement of the hip (or both hips) on clinical evaluation, persisting for more than six weeks [30,31]. All patients were referred by pediatric rheumatologists from the referential center for pediatric rheumatology. Children with diagnoses other than arthritis were excluded. None of the patients had an intraarticular corticosteroid injection.

Parents of all patients gave informed consent to take part in the study. The study was performed in accordance with the Declaration of Helsinki and was approved by the local ethics committee (KBT-3/5/2018).

Collected clinical data included age, sex, information on which hip was affected (unilaterally or bilaterally) and the final diagnosis.

### 2.2. MRI Protocol and Interpretation of Imaging Features

Non-contrast MRI examinations were performed on both hips on a 1,5 Tesla system (Siemens Avanto) in a dedicated pelvis coil. Patients were examined in a supine position. No sedation was used.

The following sequences were used: coronal T1-weighted (w) and T2 Turbo Inversion Recovery Magnitude (TIRM), axial T2w, T2 TIRM and Proton Density (PD) with fat saturation (FS), sagittal PD and axial oblique PD FS. Slice thickness was 3 mm, gap 0.6 mm.

The images were independently evaluated and scored by two radiologists (MO and IS), both with 15 years of experience in musculoskeletal imaging (10 years in rheumatological centre), blinded to clinical, laboratory data and final diagnoses. Cohen’s kappa coefficient describing inter-rater variability was calculated for each evaluated lesion. Kappa values below 0.20 were considered poor agreement, 0.21–0.40 fair, 0.41–0.60 moderate, 0.61–0.80 good and 0.81–1.00 very good [32]. In case of divergent opinions, the final diagnosis was established by consensus.

Hip MRIs were evaluated with the aim of identifying the number of active, destructive and developmental lesions that were all presented in Table 1. Definitions of lesions were adopted from the European Society of Musculoskeletal Radiology (ESSR) recommendation paper for the use of MRI in musculoskeletal rheumatic diseases [33]. Briefly, joint effusions are hyperintense on T2 and PDw images, hypointense on T1w images [33]. Synovial thickening in the active stage indicating active synovitis, shows high signal intensity on T2w, T2 FS, PD FS and T2 TIRM/STIR (Short Tau Inversion Recovery) sequences but lower than high signal from effusions in these sequences. BME in the course of the inflammation is seen as a hyperintense area on T2 and PDw images, best visualized by T2 FS or STIR/TIRM sequences, hypointense on T1w images [33]. Enthesis is hyperintense on T2 and PDw images, best visualized by T2 FS or STIR/TIRM sequences and is hypointense on T1w images. The bony part of an enthesis may show BME [33]. Bone erosions are sharply marginated trabecular bone defects with disrupted cortical bone continuity, seen in at least two planes, with low signal intensity on T1-w images. Intraosseous cysts present as high signal intensity foci on T2-w images and low signal intensity on T1-w images and they are better delineated compared with ill-defined areas of BME [33].

All active, destructive and developmental lesions were then scored and the MRI scoring system is presented in Table 1.

In addition, other inflammatory features in the pelvis were reported, within the field of view, such as sacroiliitis, pubitis and involvement of ischiopubic synchondrosis.

### 2.3. Statistical Analysis

To identify if MRI allows hip arthritis in JIA to be differentiated from hip arthralgia of unknown etiology firstly, the MRI lesions were presented as numbers and percentages. The chi-squared test (or a chi-squared test with Yates’ correction when the expected values were <5) was used for comparisons between groups. The receiver operating characteristic (ROC) curve analysis was used to verify the discriminant ability of the MRI summarized score. Sensitivity, specificity, positive predictive value (PPV) and negative predictive value (NPV) of the created diagnostic test were calculated. The statistical significance was established at *p* < 0.05. Statistical analyses were performed using Statistica v.13.1 (Dell Inc 2016, Tulsa, OK, USA).

## 3. Results

This retrospective observational study included 97 children with clinically suspected arthritis in whom non-contrast MRI of the hips was performed from 2016 to 2019. The median age of the patients was 14 years (range: 4–16 years) with a slight male predominance (52 male; 45 female).

Among the 97 included patients, JIA was confirmed in 73 (75%): 31 had oligoarthritis, 13 had the enthesitis-related arthritis subtype, 10 had polyarthritis, three had psoriatic arthritis, three had systemic JIA and 13 had undifferentiated JIA. In the remaining 24 (25%) patients, JIA was excluded and arthralgia of unknown etiology was diagnosed.

MRI showed at least one lesion in 95 patients (98%). Abnormalities in right hips were seen in five children, in left hips in six children and both hips were affected in the remaining 84 patients. Only two children were lesions free. Table 2 shows the MRI scoring results in the JIA group and the non-JIA group, divided into the left and right hips. Table 3 presents the frequency of MRI features in the compared groups.

**Figure 1:**
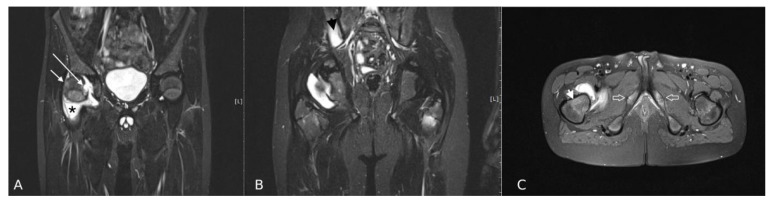
MRI of the hips in a 10-year-old boy with JIA: (**A**,**B**) Coronal T2 TIRM, (**C**) Axial T2 TIRM images. Right hip: joint effusion (stage 3), synovitis (stage 1) (asterisk), BME in femoral head (stage 1) (white short arrow), BME in the neck of femur (stage 2) (arrowhead on “C”), erosion in the femoral head (white long arrow). Left hip: BME in the femoral neck (stage 2). Right sided sacroiliitis (arrowhead on “B”). Involvement of ischiopubic synchondrosis bilaterally (empty arrows on “C”).

**Figure 2:**
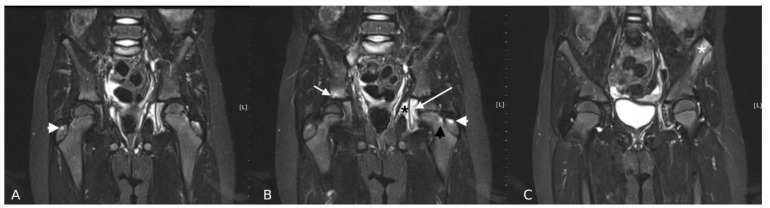
MRI of the hips in a 7-year-old girl with JIA. (**A**–**C**) Coronal T2 TIRM images. Right hip: BME in the greater Table 1. (arrowhead) and in the acetabulum (stage 1) (white short arrow). Left hip: BME in the greater trochanter (stage 1) (white arrowhead), in the acetabulum (stage 2) and triradiate cartilage (stage 1) (white long arrow) and in the femoral neck (stage 1) (black arrowhead). Obturator internus myositis (stage 1) (black asterisk). Left anterior superior iliac spine (ASIS) enthesitis (white asterisk). Right sided sacroiliitis.

Effusion was the most frequent abnormality (Figure 1). Almost all children had joint effusion of stage 1, 2, or 3 (*n* = 95, 98%). Stage 1 effusion was most frequently identified in all children, regardless of diagnosis (i.e., 90% vs. 92% in the JIA vs. non-JIA group, respectively); thus, it had no discriminatory value (*p* = 0.825). In contrast, the incidence of stage 2 and 3 effusion was higher in JIA patients than in the non-JIA group (25% vs. 4%, respectively), with stage 3 effusion only present in the JIA group. The next most common abnormalities were BME in the femoral head, synovitis, enthesitis and BME in the neck of the femur (Table 2 and Table 3) (Figure 1 and Figure 2).

All lesions were more common in the JIA group than the non-JIA group; however, none of them was significantly more common in JIA vs. non-JIA group. Several lesions were approaching the level of significance *p*< 0.05: in JIA group effusion 2 or 3 were seen in 23% vs. 4% (*p* = 0.074), BME within femoral head in 22% vs. 4% (*p* = 0.094) and BME of the femoral neck in 16% vs. 0% (*p* = 0.078). BME within the neck, acetabulum and greater trochanter, stage 2 BME in the head of the femur, stage 3 effusion, as well as synovitis, myositis and triradiate cartilage involvement were exclusively seen in the JIA group (Table 2 and Table 3). Destructive lesions were only diagnosed in JIA group and most frequent were: chondromalacia, followed by JSN, cysts and erosions (Table 2 and Table 3). None of the 97 patients had protrusio acetabuli, ankylosis, physis involvement, or secondary osteoarthritis features, such as sclerotization and osteophytes. Single cases of AVN and bone remodeling (widening of the femoral neck) were seen in both groups (Table 2 and Table 3).

Sacroiliitis was observed in four (5%) children with JIA and in one (4%) without a final diagnosis of JIA (*p* = 0.780) (Figure 1 and Figure 2). Pubitis was present in two children with JIA (3%) and in none from the non-JIA group (*p* = 0.993).

There was a very good interobserver agreement for scoring all active and destructive lesions, except for six features: BME in the head of femur (two cases, kappa 0.94), BME in the neck of the femur (two cases, kappa 0.91), joint effusion (one case, kappa 0.99) and gluteus medius tendon enthesitis (one case, kappa 0.98). In all 6 cases of interobserver disagreements the border scores were provided, including: score 0 was given by one observer and score 1 by the second in case of discrete BME in the femoral head, score 1 and 2 when BME was around 50% of femoral head and score 0 and 1 in case of small joint fluid, that was interpreted as physiology by one observer and as a small effusion by the second.

**Figure 3:**
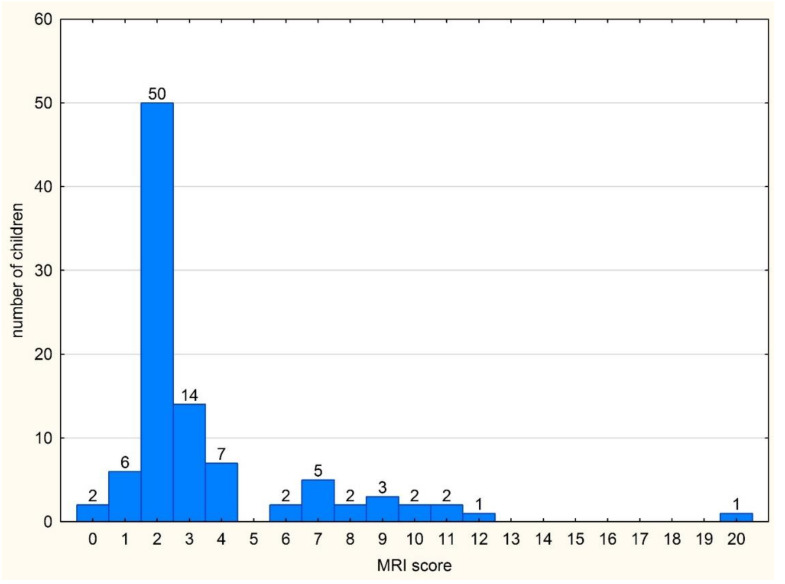
The frequency of individual MRI scores in the whole group of patients (*n* = 97).

Due to the fact that any single lesion was significantly more common in children with JIA compared to the non-JIA group, we decided to build a scoring. The MRI summarized score was the sum of scorings of all hip lesions in an individual patient. The score included all 24 lesions presented in Table 1. The frequencies of MRI scores in the whole group of patients are presented in Figure 3.

The median interquartile range (IQR) of MRI summarized score was significantly higher in patients with a final diagnosis of JIA compared to the non-JIA group: median (IQR) 3 (2–5) vs. 2 (2–2), respectively, *p* = 0.002.

**Figure 4:**
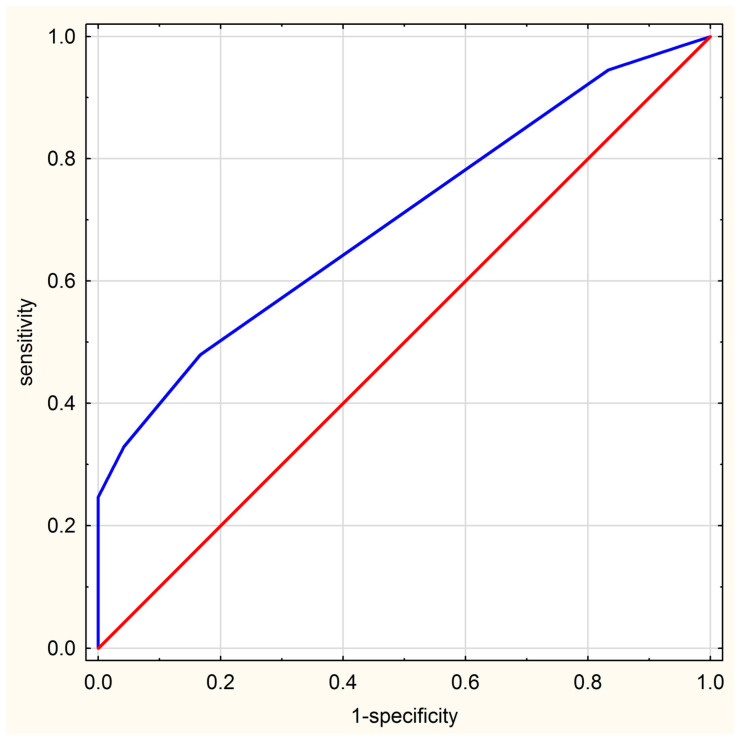
ROC curve of MRI score.

Table 4 shows the properties of diagnostic tests based on MRI summarized score values. At the cut-off point set at 3, the MRI summarized score has a sensitivity of 48% and a specificity of 83%. However, if a patient has an MRI summarized score of 6, the specificity of the method increases to 100%, but the sensitivity drops to 25%. The area under the curve (AUC) for this diagnostic test amounted to 0.704 (95% confidence interval (CI): 0.595–0.813), indicating good discriminative ability between JIA and non-JIA (Figure 4).

## 4. Discussion

MRI allows hip arthritis in JIA to be differentiated from hip arthralgia of unknown etiology with good specificity, thus, may be helpful in confirming the diagnosis of JIA. Despite limited sensitivity, the specificity of MRI is high and for the summarized score 6 it comes to 100%.

Over the last decade, there has been an increasing move towards earlier and more aggressive treatment of JIA with methotrexate and biological therapy in the hope of preventing joint damage [8,34]. However, in the context of coxitis, decisions to escalate treatment may be limited because of the difficulties in confirming arthritis by clinical examination [4,11,16,34,35,36].

Contrary to wrist and knee joints, only a few studies have examined the role of MRI in evaluating hip disease in JIA [5,6,8,11]. Our findings indicate that MRI is more useful in confirming hip arthritis in JIA than clinical assessment. Likewise, a higher sensitivity of MRI over clinical diagnosis was reported by El-Azeem et al. [4] and Nistala et al. [11].

The most common abnormality was stage 1 effusion, in both JIA-confirmed and non-JIA groups (90% vs. 92%, respectively) and had no discriminatory value (*p* = 0.825). Stage 1 effusion, which was a trace of fluid (thickness ≤ 2 mm) was also used in other researchers’ staging systems for hip joint [4,27,37]. At the same time, such small amounts of fluid in the hip were detected in healthy joints and was regarded as physiologic [22,37]. In a study performed on adult patients with normal and ischemic hips [37], stage 1 fluid was seen in the majority of asymptomatic hips (95%), whereas stage 2 fluid (surrounding the femoral neck) was present in only four hips (5%) and none had stage 3 effusion.

Synovitis, which is a hallmark of joint inflammation, was significantly more common in the JIA group than in the non-JIA group (15 vs. 0 patients; *p* = 0.037). We used a score of 1 if any visible synovium was observed in the hip on non-contrast MRI, regardless of its thickness. This classification is in contrast to most other MRI studies on different joints in JIA, where an abnormal synovium was defined as a thickness of ≥2 mm [2,4,8,27], or the thickness was not specified [5,6,11]. We chose to include any visible synovium as there is limited data on the normal values for synovium thickness. In healthy individuals, the synovium comprises an intimal layer, which is 20–40 µm thick in cross-section and an areolar subintima, which can be up to 5 mm in thickness [38]. Meanwhile, El-Azeem et al. [4] reported that the synovial thickness in clinically active juvenile hips was 3.56 ± 1.81 mm, indicating that a synovial thickness of less than 2 mm layer can be pathologic. Therefore, more studies are needed to define normal and pathologic values for the synovium on MRI.

We did not use an intravenous contrast agent in this study, as it not only prolongs the examination time, increases costs and patient discomfort and has an added (albeit rarely in MRI) risk of allergic reactions. The most important risks in pediatric JIA patients are those connected with the accumulation of the contrast agent in the kidneys and basal ganglia, even after a prolonged period [20,21]. As JIA often involves numerous joints and contrast agent must be injected for multiple examinations (i.e., for both diagnosis and monitoring), it can cause a significant burden. Hemke et al. [2] found unenhanced MRI of the knee joint of low sensitivity (0.62) than Gadolinium-enhanced MRI for the detection of synovial hypertrophy, but specificity remained high (0.97). On the other hand, Nusman et al. found enhancing synovium in 52% of the knees in healthy children [39]. The current study showed that non-contrast MRI of hip joints is satisfactory in discriminating between JIA and non-JIA patients and—with the use of the proposed MRI summarized score—may diagnose JIA with a specificity up to 100%. These results are promising; however, more studies focused on the hip joint are needed to confirm the findings.

Features of advanced hip damage (such as cysts, erosions, chondromalacia and JSN) were only observed in the JIA group in three patients. The disease duration in these patients was nine months in one patient and nine years in the other two. AVN was observed in four children: three in the JIA group and one in the non-JIA group. AVN is more common in older juveniles [1] and, indeed, our patients were 13 years (two patients) and 16 years (two patients). Only one patient was taking corticosteroids for three months before MRI. This is in agreement with observations that AVN in rheumatic patients is not exclusively related to steroid intake but may also result from the disease itself (i.e., the released cytokines or vasculopathy) [40].

As for now, the role of imaging is limited for the diagnosis of JIA, because it is based on the clinical findings and laboratory data. However, the recent classification criteria proposed by Pediatric Rheumatology International Trials Organization (PRINTO) already recommend the use of imaging to evaluate sacroiliitis [30,41]. For the hip joint, which is also difficult to assess by clinical examination, when radiographs and ultrasound findings are equivocal, an MRI could be performed to confirm the diagnosis and for narrowing the differential diagnoses [29]. The proposed scoring may have practical applicability in predicting JIA involvement. Based on results it can be concluded that a child who has the MRI summarized score of at least 6 has a high probability of suffering from JIA. In future studies, these values may be modified and another cut-off value might be proposed based on the frequency of diagnosed lesions; some features might be deleted whereas others, e.g., those at the border of significance (effusion, BME) may become significant.

The main strength of this study is the inclusion of a large number of patients. All children were referred from a single pediatric rheumatology center, indicating the clinical examination was reliable. Another benefit of this study is a large number of MRI features (24 altogether) included in the scoring system, which improves statistical characteristics of proposed diagnostic test. Short time needed to fill the scoring sheet (c.a. 2 min) is also encouraging.

The main limitation of this study was omitting the contrast agent. This was a conscious decision reflecting the authors’ everyday practice. Moreover, the study did not include a healthy control group and only hip joints in children without a final diagnosis of JIA served for comparison. While it is hard to get pediatric and adolescent control data, these would be absolutely required for future.

## 5. Conclusions

MRI is useful in confirming hip inflammatory features in children with clinically suspected arthritis. Patients with JIA develop more lesions and are more advanced than children with hip arthralgia of unknown origin. The MRI allows hip arthritis in JIA to be differentiated from hip arthralgia of unknown etiology with good specificity and, thus, may be helpful in confirming the diagnosis of JIA. Future studies are needed to validate this new scoring system and to further investigate for clinical practice.

## Figures and Tables

**Table 1:** MRI scoring system.

	MRI Feature	Scoring
1	Effusion	0–3 0: no fluid; 1: trace of fluid and maximum thickness of ≤2 mm 2: continual effusion and thickness >2 mm and ≤5 mm 3: effusion with distension of capsule and thickness >5 mm
2	BME head	0–2 0: BME not seen 1: BME up to 50% of the head width 2: BME > 50% of the head width
3	BME neck	0–2 0: BME not seen 1: BME up to 50% of the neck width 2: BME > 50% of the neck width
4	BME acetabulum	0–2 0: BME not seen 1: BME up to 50% of the acetabular width 2: BME > 50% of the acetabular width
5	BME greater trochanter	0–1
6	Synovitis	0–1 0: synovium not visible 1: synovium visible regardless the thickness
7	Bursitis	0–1
8	Enthesitis	0–1
9	Tendinitis	0–1
10	Myositis	0–1
11	Triradiate cartilage involvement	0–1
12	Physeal involvment	0-1
13	Cyst femoral head	0–1
14	Cyst acetabulum	0–1
15	Erosion femoral head	0–1
16	Erosion acetabulum	0–1
17	Chondromalacia	0–1
18	Joint space narrowing	0–1
19	Protrusio acetabuli	0–1
20	Ankylosis	0–1
21	Sclerotization	0–1
22	Osteophytes	0–1
23	Avascular necrosis	0–1
24	Bone remodeling	0–1

**Table 2:** MRI scoring results for left and right hips.

	MRI Lesions and Scorings	JIA Confirmed Group *n* = 73		Non-JIA Group *n* = 24	
		Number of Lesions		Number of Lesions	
		Left Hip	Right Hip	Left Hip	Right Hip
1	Effusion				
	0	6	7	3	3
	1	59	54	20	20
	2	4	6	1	1
	3	4	6	0	0
2	BME head				
	0	62	66	24	23
	1	9	3	0	1
	2	2	4	0	0
3	BME neck				
	0	66	67	24	24
	1	4	4	0	0
	2	3	2	0	0
4	BME acetabulum				
	0	70	68	24	24
	1	3	3	0	0
	2	0	2	0	0
5	BME greater trochanter				
	0	66	69	24	24
	1	7	4	0	0
6	Synovitis				
	0	67	64	24	24
	1	6	9	0	0
7	Bursitis				
	0	68	72	23	24
	1	5	1	1	0
8	Enthesitis				
	0	65	66	24	24
	1	8	7	0	0
9	Tendinitis				
	0	73	73	24	24
	1	0	0	0	0
10	Myositis				
	0	72	71	24	24
	1	1	2	0	0
11	Triradiate cartilage involvement				
	0	72	72	24	24
	1	1	1	0	0
12	Physeal involvment				
	0	73	73	24	24
	1	0	0	0	0
13	Cyst femoral head				
	0	71	72	24	24
	1	2	1	0	0
14	Cyst acetabulum				
	0	72	72	24	24
	1	1	1	0	0
15	Erosion femoral head				
	0	73	71	24	24
	1	0	2	0	0
16	Erosion acetabulum				
	0	72	73	24	24
	1	1	0	0	0
17	Chondromalacia				
	0	68	71	24	24
	1	5	2	0	0
18	Joint space narrowing				
	0	71	70	24	24
	1	2	3	0	0
19	Protrusio acetabuli				
	0	73	73	24	24
	1	0	0	0	0
20	Ankylosis				
	0	73	73	24	24
	1	0	0	0	0
21	Sclerotization				
	0	73	73	24	24
	1	0	0	0	0
22	Osteophytes				
	0	73	73	24	24
	1	0	0	0	0
23	Avascular necrosis				
	0	71	72	24	23
	1	2	1	0	1
24	Bone remodeling				
	0	72	72	24	23
	1	1	1	0	1

**Table 3:** Frequency of MRI features in the compared groups.

	MRI Feature and Scoring at Least in One Hip	JIA Confirmed Group	Non-JIA Group	*p* *
1	Effusion			
	0	12 (16%)	5 (21%)	0.856
	1	66 (90%)	22 (92%)	0.825
	2	8 (11%)	1 (4%)	0.555
	3	10 (14%)	0 (0%)	0.127
	2 or 3	17 (23%)	1 (4%)	0.074
2	BME head			
	0	71 (97%)	24 (100%)	0.993
	1	11 (15%)	1 (4%)	0.294
	2	6 (8%)	0 (0%)	0.336
	1 or 2	16 (22%)	1 (4%)	0.094
3	BME neck			
	0	72 (99%)	24 (100%)	0.556
	1	8 (11%)	0 (0%)	0.206
	2	5 (7%)	0 (0%)	0.433
	1 or 2	12 (16%)	0 (0%)	0.078
4	BME acetabulum			
	0	72 (99%)	24 (100%)	0.556
	1	6 (8%)	0 (0%)	0.336
	2	2 (3%)	0 (0%)	0.993
	1 or 2	7 (10%)	0 (0%)	0.263
5	BME greater trochanter			
	0	70 (96%)	24 (100%)	0.742
	1	8 (11%)	0 (0%)	0.206
6	Synovitis			
	0	73 (100%)	24 (100%)	1
	1	15 (21%)	0 (0%)	0.037
7	Bursitis			
	0	72 (99%)	24 (100%)	0.556
	1	5 (7%)	1 (4%)	0.988
8	Enthesitis			
	0	72 (99%)	24 (100%)	0.556
	1	14 (19%)	0 (0%)	0.047
9	Tendinitis			
	0	73 (100%)	24 (100%)	1
	1	0 (0%)	0 (0%)	1
10	Myositis			
	0	73 (100%)	24 (100%)	1
	1	3 (4%)	0 (0%)	0.742
11	Triradiate cartilage involvement			
	0	73 (100%)	24 (100%)	1
	1	1 (1%)	0 (0%)	0.556
12	Physeal involvment			
	0	73 (100%)	24 (100%)	1
	1	0 (0%)	0 (0%)	1
13	Cyst femoral head			
	0	73 (100%)	24 (100%)	1
	1	3 (4%)	0 (0%)	0.742
14	Cyst acetabulum			
	0	73 (100%)	24 (100%)	1
	1	2 (3%)	0 (0%)	0.993
15	Erosion femoral head			
	0	73 (100%)	24 (100%)	1
	1	2 (3%)	0 (0%)	0.993
16	Erosion acetabulum			
	0	73 (100%)	24 (100%)	1
	1	1 (1%)	0 (0%)	0.556
17	Chondromalacia			
	0	73 (100%)	24 (100%)	1
	1	7 (10%)	0 (0%)	0.263
18	Joint space narrowing			
	0	72 (99%)	24 (100%)	0.556
	1	4 (5%)	0 (0%)	0.562
19	Protrusio acetabuli			
	0	73 (100%)	24 (100%)	1
	1	0 (0%)	0 (0%)	1
20	Ankylosis			
	0	73 (100%)	24 (100%)	1
	1	0 (0%)	0 (0%)	1
21	Sclerotization			
	0	73 (100%)	24 (100%)	1
	1	0 (0%)	0 (0%)	1
22	Osteophytes			
	0	73 (100%)	24 (100%)	1
	1	0 (0%)	0 (0%)	1
23	Avascular necrosis			
	0	73 (100%)	24 (100%)	1
	1	3 (4%)	1 (4%)	0.562
24	Bone remodeling			
	0	72 (99%)	23 (96%)	0.556
	1	1 (1%)	1 (4%)	0.993

* The *p*-value refers to a comparison of the frequency of the score listed in the first column to the frequency of all other scores for that feature, for example (2 or 3) for effusion is the comparison of scores (2 or 3) vs. (1 or 0). Values of one feature do not add up to 100%, because the scores relate to assessment in at least one hip in each child (as noted in the column header).

**Table 4:** Diagnostic value of the summarized MRI score as a predictor of JIA.

MRI Summarised Score	JIA	Non-JIA	True Pos.	False Pos.	False Neg.	True Neg.	Sens.	Spec.	PPV	NPV
20	1	0	1	0	72	24	0.014	1.000	1.000	0.250
12	1	0	2	0	71	24	0.027	1.000	1.000	0.253
11	2	0	4	0	69	24	0.055	1.000	1.000	0.258
10	2	0	6	0	67	24	0.082	1.000	1.000	0.264
9	3	0	9	0	64	24	0.123	1.000	1.000	0.273
8	2	0	11	0	62	24	0.151	1.000	1.000	0.279
7	5	0	16	0	57	24	0.219	1.000	1.000	0.296
6	2	0	18	0	55	24	0.247	1.000	1.000	0.304
4	6	1	24	1	49	23	0.329	0.958	0.960	0.319
3	11	3	35	4	38	20	0.479	0.833	0.897	0.345
2	34	16	69	20	4	4	0.945	0.167	0.775	0.500
1	3	3	72	23	1	1	0.986	0.042	0.758	0.500
0	1	1	73	24	0	0	1.000	0.000	0.753	
